# Supplementary material for: Causal Relationship between Aging and Anorexia Nervosa: A White-Matter-Microstructure-Mediated Mendelian Randomization Analysis
Source: Biomedicines. 2024 Aug 16;12(8):1874. doi: 10.3390/biomedicines12081874 (PMC11351342; doi:10.3390/biomedicines12081874)
Supplement: Supplementary file 1 [file biomedicines-12-01874-s001.zip › Supplementary Figure/Figure S4/Singe SNP plot of MR analysis for TL to ubm-b-2047.pdf]

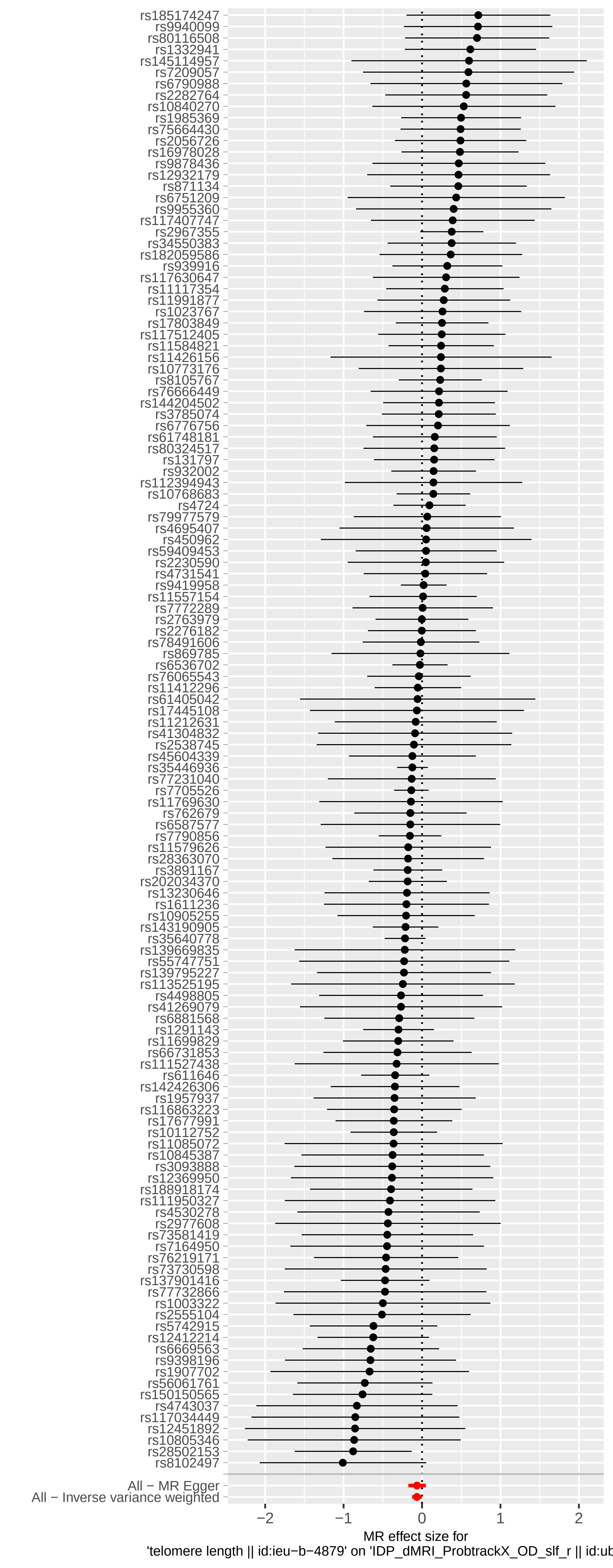
rs185174247
rs9940099
rs80116508
rs1332941
rs145114957
rs7209057
rs6790988
rs2282764
rs10840270
rs1985369
rs75664430
rs2056726
rs16978028
rs9878436
rs12932179
rs871134
rs6751209
rs9955360
rs117407747
rs2967355
rs34550383
rs182059586
rs939916
rs117630647
rs11117354
rs11991877
rs1023767
rs17803849
rs117512405
rs11584821
rs11426156
rs10773176
rs8105767
rs76666449
rs144204502
rs3785074
rs6776756
rs61748181
rs80324517
rs131797
rs932002
rs112394943
rs10768683
rs4724
rs79977579
rs4695407
rs450962
rs59409453
rs2230590
rs4731541
rs9419958
rs11557154
rs7772289
rs2763979
rs2276182
rs78491606
rs869785
rs6536702
rs76065543
rs11412296
rs61405042
rs17445108
rs11212631
rs41304832
rs2538745
rs45604339
rs35446936
rs77231040
rs7705526
rs11769630
rs762679
rs6587577
rs7790856
rs11579626
rs28363070
rs3891167
rs202034370
rs13230646
rs1611236
rs10905255
rs143190905
rs35640778
rs139669835
rs55747751
rs139795227
rs113525195
rs4498805
rs41269079
rs6881568
rs1291143
rs11699829
rs66731853
rs111527438
rs611646
rs142426306
rs1957937
rs116863223
rs17677991
rs10112752
rs11085072
rs10845387
rs3093888
rs12369950
rs188918174
rs111950327
rs4530278
rs2977608
rs73581419
rs7164950
rs76219171
rs73730598
rs137901416
rs77732866
rs1003322
rs2555104
rs5742915
rs12412214
rs6669563
rs9398196
rs1907702
rs56061761
rs150150565
rs4743037
rs117034449
rs12451892
rs10805346
rs28502153
rs8102497
All − MR Egger
All − Inverse variance weighted
−2
−1
0
1
2
MR effect size for
'telomere length || id:ieu−b−4879' on 'IDP_dMRI_ProbtrackX_OD_slf_r || id:ub
